# Gene Tree Labeling Using Nonnegative Matrix Factorization on Biomedical Literature

**DOI:** 10.1155/2008/276535

**Published:** 2008-04-09

**Authors:** Kevin E. Heinrich, Michael W. Berry, Ramin Homayouni

**Affiliations:** ^1^Department of Electrical Engineering and Computer Science, University of Tennessee, Knoxville, TN 37996-3450, USA; ^2^Department of Biology, University of Memphis, Memphis, TN 38152-3150, USA

## Abstract

Identifying functional groups of genes is a challenging problem for biological applications. Text mining approaches can be used to build hierarchical clusters or trees from the information in the biological literature. In particular, the nonnegative matrix factorization (NMF) is examined as one approach to label hierarchical trees. A generic labeling algorithm as well as an evaluation technique is proposed, and the effects of different NMF parameters with regard to convergence and labeling accuracy are discussed. The primary goals of this study are to provide a qualitative assessment of the NMF and its various parameters and initialization, to provide an automated way to classify biomedical data, and to provide a method for evaluating labeled data assuming a static input tree. As a byproduct, a method for generating *gold standard* trees is proposed.

## 1. INTRODUCTION

High-throughput techniques in genomics, proteomics, and related biological fields generate large amounts of data that enable researchers to examine biological systems from a global perspective. Unfortunately, however, the sheer mass of information available is overwhelming, and data such as gene expression profiles from DNA microarray analysis can be difficult to understand fully even for domain experts. Additionally, performing these experiments in the lab can be expensive with respect to both time and money.

In recent years, biological literature repositories have become an alternative data source to examine phenotype. Many of the online literature sources are manually curated, so the annotations assigned to articles are subjectively assigned in an imperfect and error-prone manner. Given the time required to read and classify an article, automated methods may help increase the annotation rate as well as improve existing annotations.

A recently developed tool that may help improve annotation as well as identify functional groups of genes is the Semantic Gene Organizer (SGO). SGO is a software environment based upon latent semantic indexing (LSI) that enables researchers to view groups of genes in a global context as a hierarchical tree or dendrogram [1]. The low-rank approximation provided by LSI (for the original term-to-document associations) exposes latent relationships so that the resulting hierarchical tree is simply a visualization of those relationships that are reproducible and easily interpreted by biologists. Homayouni et al. [2] have shown that SGO can identify groups of related genes more accurately than term co-occurrence methods. LSI, however, is based upon the singular value decomposition (SVD) [3], and since the input data for SGO is a nonnegative matrix of weighted term frequencies, the negative values prevalent in the basis vectors of the SVD are not easily interpreted.

On the other hand, the decomposition produced by the recently popular nonnegative matrix factorization (NMF) can be readily interpreted. Paatero and Tapper [4] were among the first researchers to investigate this factorization, and Lee and Seung [5] demonstrated its use for both text mining and image analysis. NMF is generated by an iterative algorithm that preserves the nonnegativity of the original data; the factorization yields a low-rank, parts-based representation of the data. In effect, common themes present in the data can be identified simply by inspecting the factor matrices. Depending on the interpretation, the factorization can induce both clustering and classification. If NMF can accurately model the input data, it can be used to both classify data and perform pattern recognition tasks [6]. Within the context of SGO, this means that the groups of genes presented in the hierarchical trees can be assigned labels that identify common attributes of protein function.

The interpretability of NMF, however, comes at a price. Namely, convergence and stability are not guaranteed, and many variations have been proposed [5], requiring different parameter choices. The goals of this study are (1) to provide a qualitative assessment of the NMF and its various parameters, particularly as they apply to the biomedical context, (2) to provide an automated way to classify biomedical data, and (3) to provide a method for evaluating labeled data assuming a static input tree. As a byproduct, a method for generating “gold standard” trees is proposed.

## 2. METHODS

As outlined in [7], hierarchical trees can be constructed for a given group of genes. Once those trees are formed, techniques that label the interior nodes of those trees can be examined.

### 2.1. Nonnegative matrix factorization

Given an *m* × *n* nonnegative matrix *A* = [*a_ij_*], where each entry *a_ij_* denotes the term weight of token *i* in gene document *j*, the rows of *A* represent term vectors that show how terms are distributed across the entire collection. Similarly, the columns of *A* show which terms are present within a gene document. Consider the 24 × 9 term-by-document matrix *A* in Table 1 derived from the sample document collection [7] in Table 2. Here, log-entropy term weighting [8] is used to define the relative importance of term *i* for document *j*. Specifically, *a_ij_* =*a_ijgi_*, where

(1) $$\begin{array}{ll} l_{ij} & =\log_{2}\left(1+f_{ij}\right),\\ g_{i} & =1+\left(\frac{\sum_{j}\left(p_{ij}\log_{2}p_{ij}\right)}{\log_{2}n}\right), \end{array}$$

*f_ij_* is the frequency of token *i* in document *j*, and *p_ij_* =*f_ij_*/∑*_j_f_ij_* is the probability of token *i* occurring in document *j*. By design, tokens that appear less frequently across the collection but more frequently within a document will be given higher weight. That is, distinguishing tokens will tend to have higher weights assigned to them, while more common tokens will have weights closer to zero.

If NMF is applied to the sample term-document matrix in Table 1, one possible factorization is given in Tables 3 and 4; the approximation to the term-document matrix generated by mutliplying *W* × *H* is given in Table 5. The top-weighted terms for each feature are presented in Table 6. By inspection, the sample collection has features that represent *leukemia*, *alcoholism*, *anxiety*, and *autism*. If each document and term is assigned to its most dominant feature, then the original term-document matrix can be reorganized around those features. The restructured matrix typically resembles a block diagonal matrix and is given in Table 7.

NMF of *A* is based on an iterative technique attempts to find two nonnegative factor matrices, *W* and *H*, such that

(2) A≈WH,

where *W* and *H* are *m* × *k* and *k* × *n* matrices, respectively. Typically, *k* is chosen so that *k* ≪ min(*m*, *n*). The optimal choice of *k* is problem-dependant [9]. This factorization minimizes the squared Euclidean distance objective function [10]

(3) $${∥A-WH∥}_{F}^{2}=\underset{ij}{\sum }{\left(A_{ij}-{\left(WH\right)}_{ij}\right)}^{2}.$$

Minimizing the objective (or cost) function is convex in either *W* or *H*, but not both variables together. As such, finding global minima to the problem is unrealistic—however, finding several local minima is within reason. Also, for each solution, the matrices *W* and *H* are not unique. This property is evident when examining *WD* *D* ^−1^ *H* for any nonnegative invertible matrix *D* [11].

The goal of NMF is to approximate the original term-by-gene document space as accurately as possible with the factor matrices *W* and *H*. As noted in [12], the singular value decomposition (SVD) produces the optimal rank-*k* approximation with respect to the Frobenius norm. Unfortunately, this optimality frequently comes at the cost of negative elements. The factor matrices of NMF, however, are strictly nonnegative which may facilitate direct interpretability of the factorization. Thus, although an NMF approximation may not be optimal from a mathematical standpoint, it may be sufficient and yield better insight into the dataset than the SVD for certain applications.

Upon completion of NMF, the factor matrices *W* and *H* will, in theory, approximate the original matrix *A* and yet contain some valuable information about the dataset in question. As presented in [10], if the approximation is close to the original data, then the factor matrices can uncover some underlying structure within the data. To reinforce this, *W* is commonly referred to as the *feature matrix* containing *feature vectors* that describe the themes inherent within the data while *H* can be called a *coefficient matrix* since its columns describe how each document spans each feature and to what degree.

Currently, many implementations of NMF rely on random nonnegative initialization. As NMF is sensitive to its initial seed, this obviously hinders the reproducibility of results generated. Boutsidis and Gallopoulos [13] propose the nonnegative double singular value decomposition (NNDSVD) scheme as a possible remedy to this concern. NNDSVD aims to exploit the SVD as the optimal rank-*k* approximation of *A*. The heuristic overcomes the negative elements of the SVD by enforcing nonnegativity whenever encountered and by iteratively approximating the outer product of each pair of singular vectors. As a result, some of the properties of the data are preserved in the initial starting matrices *W* and *H*. Once both matrices are initialized, they can be updated using the multiplicative rule [10]:

(4) $$\begin{array}{ll} H_{cj} & \leftarrow H_{cj}\frac{{\left(W^{T}A\right)}_{cj}}{{\left(W^{T}WH\right)}_{cj}},\\ W_{ic} & \leftarrow W_{ic}\frac{{\left(AH^{T}\right)}_{ic}}{{\left(WHH^{T}\right)}_{ic}}. \end{array}$$

### 2.2. Labeling algorithm

Latent semantic indexing (LSI), which is based on the SVD, can be used to create a global *picture* of the data automatically. In this particular context, hierarchical trees can be constructed from pairwise distances generated from the low-rank LSI space. Distance-based algorithms such as FastME can create hierarchies that accurately approximate distance matrices in *O*(*n* ^2^) time [14]. Once a tree is built, a labeling algorithm can be applied to identify branches of the tree. Finally, a “gold standard” tree and a standard performance measure that evaluates the quality of tree labels must be defined and applied.

Given a hierarchy, few well-established automated labeling methods exist. To apply labels to a hierarchy, one can associate a weighted list of terms with each taxon. Once these lists have been determined, labeling the hierarchy is simply a matter of recursively inheriting terms up the tree from each child node; adding weights of shared terms will ensure that more frequently used terms are more likely to have a larger weight at higher levels within the tree. Intuitively, these terms are often more general descriptors.

This algorithm is robust in that it can be slightly modified and applied to any tree where a ranked list can be applied to each taxon. For example, by querying the SVD-generated vector space for each document, a ranked list of terms can be created for each document and the tree labeled accordingly. As a result, assuming the initial ranking procedure is accurate, any ontological annotation can be enhanced with terms from the text it represents.

To create a ranked list of terms from NMF, the dominant coefficient *H_ij_* in *H* is extracted for document *j*. The corresponding feature *W_i_* is then scaled by *H_ij_* and assigned to the taxon representing document *j*, and the top 100 terms are chosen to represent the taxon. This method can be expanded to incorporate branch length information, thresholds, or multiple features.

### 2.3. Recall measure

Once labelings are produced for a given hierarchical tree, a measure of “goodness” must be calculated to determine which labeling is the “best.” When dealing with simple return lists of documents that can be classified as either relevant or not relevant to a user's needs, information retrieval (IR) methods typically default to using precision and recall to describe the performance of a given retrieval system. Precision is the ratio of relevant returned items to total number of returned items, while recall is the percentage of relevant returned items with respect to the total number of relevant items. Once a group of words is chosen to label an entity, the order of the words carries little meaning, so precision has limited usefulness in this application. When comparing a generated labeling to a “correct” one, recall is an intuitive measure.

Unfortunately in this context, one labelled hierarchy must be compared to another. Surprisingly, relatively little work has been done that addresses this problem. Kiritchenko in [15] proposed the hierarchical precision and recall measures, denoted as *hP* and *hR*, respectively. These measures take advantage of hierarchical consistency to compare two labelings with a single number. Unfortunately, condensing all the information held in a labeled tree into a single number loses some information. In the case of NMF, the effects of parameters on labeling accuracy with respect to node depth is of interest, so a different measure would be more informative. One such measure finds the average recall of all the nodes at a certain depth within the tree. To generate nonzero recall, however, common terms must exist between the labelings being compared. Unfortunately, many of the terms present in MeSH headings are not strongly represented in the text. As a result, the text vocabulary must be mapped to the MeSH vocabulary to produce significant recall.

### 2.4. Feature vector replacement

When working with gene documents, many cases exist where the terminology used in MeSH is not found within the gene documents themselves. Even though a healthy percentage of the exact MeSH terms may exist in the corpus, the term-document matrix is so heavily overdetermined (i.e., the number of terms is significantly larger than the number of documents) that expecting significant recall values at any level within the tree becomes unreasonable. This is not to imply that the terms produced by NMF are without value. On the contrary, the value in those terms is exactly that they may reveal what was previously unknown. For the purposes of validation, however, some method must be developed that enables a user to discriminate between labelings even though both have little or no recall with the MeSH-labeled hierarchy. In effect, the vocabulary used to label the tree must be controlled for the purposes of validation and evaluation.

To produce a labeling that is mapped into the MeSH vocabulary, the top *r* globally-weighted MeSH headings are chosen for each document; these MeSH headings can be extracted from the MeSH metacollection [7]. By inspection of *H*, the dominant feature associated with each document is chosen and assigned to that document. The corresponding top *r* MeSH headings are then themselves parsed into tokens and assigned to a new MeSH feature vector appropriately scaled by the corresponding coefficient in *H*. The feature vector replacement algorithm is given in Algorithm 1. Note that *m*′ is distinguished from *m* since the dictionary of MeSH headings will likely differ in size and composition from the original corpus dictionary. The number of documents, however, remains constant.

Once full MeSH feature vectors have been constructed, the tree can be labeled via the procedure outlined in [7]. As a result of this replacement, better recall can be expected, and the specific word usage properties inherent in the MeSH (or any other) ontology can be exploited.

### 2.5. Alternative labeling method

An alternative method to label a tree is to vary the parameter *k* from (2) with node depth. In theory, more pertinent and accurate features will be preserved if the clusters inherent in the NMF coincide with those in the tree generated via the SVD space. For smaller clusters and more specific terms, higher *k* should be necessary; conversely, the ancestor nodes should require smaller *k* and more general terms since they cover a larger set of genes spanning a larger set of topics. Inheritance of terms can be performed once again by inheriting common terms—however, an upper threshold of inheritance can be imposed. For example, for all the nodes in the subtree induced by a node *p*, high *k* can be used. If all the genes induced by *p* are clustered together by NMF, then all the nodes in the subtree induced by *p* will maintain the same labels. For the ancestor of *p*, a different value of *k* can be used. Although this method requires some manual curation, it can potentially produce more accurate labels.

## 3. RESULTS

The evaluation of the factorization produced by NMF is nontrivial as there is no set standard for examining the quality of basis vectors produced. In several studies thus far, the results of NMF runs have been evaluated by domain experts. For example, Chagoyen et al. [16] performed several NMF runs and then independently asked domain experts to interpret the resulting feature vectors. This approach, however, limits the usefulness of NMF, particularly in discovery-based genomic studies for which domain experts are not readily available. Here, two different automated protocols are presented to evaluate NMF results. First, the mathematical properties of the NMF runs are examined, then the accuracy of the application of NMF to hierarchical trees is scrutinized.

### 3.1. Input parameters

To test NMF, the *50TG* collection presented in [2] was used. This collection was constructed manually by selecting genes known to be associated with at least one of the following categories: (1) development, (2) Alzheimer's disease, and (3) cancer biology. Each gene document is simply a concatenation of all titles and abstracts of the MEDLINE citations cross-referenced in the mouse, rat, and human EntrezGene (formerly LocusLink) entries for each gene.

Two different NMF initialization strategies were used: the NNDSVD [17] and randomization. Five different random trials were conducted while four were performed using the NNDSVD method. Although the NNDSVD produces a static starting matrix, different methods can be applied to remove zeros from the initial approximation to prevent them from getting “locked” throughout the update process. Initializations that maintained the original zero elements are denoted NNDSVDz, while NNDSVDa, NNDSVDe, and NNDSVDme substitute the average of all elements of *A*, *ϵ*, or $\epsilon_{\text{machine}}$, respectively, for those zero elements; *ε* was set to 10^−9^ and was significantly smaller than the smallest observed value in either *H* or *W* (typically around 10^−3^), while $\epsilon_{\text{machine}}$ was the machine epsilon (the smallest positive value the computer could represent) at approximately 10^−324^. Both NNDSVDz and NNDSVDa were described previously in [13], whereas NNDSVDe and NNDSVDme are added in this study as natural extensions to NNDSVDz that would not suffer from the restrictions of locking zeros due to the multiplicative update. The parameter *k* was assigned the values of 2, 4, 6, 8, 10, 15, 20, 25, and 30.

Each of the NMF runs iterated until it reached 1,000 iterations or a stationary point in both *W* and *H*. That is, at iteration *i*, when ||*W*_*i*−1_ − *W_i_*||_*F*_ < *τ* and ||*H*_*i*−1_ − *H_i_*||_*F*_ < *τ*, convergence is assumed. The parameter *τ* was set to 0.01. Since convergence is not guaranteed under all constraints, if the objective function increased between iterations, the factorization was stopped and assumed not to converge. Log-entropy term-weighting scheme (see [8]) was used to generate the original token weights for each collection.

### 3.2. Relative error and convergence

The SVD produces the mathematically optimal low-rank approximation of any matrix with respect to the Frobenius norm, and for all other unitarily-invariant matrix norms. Whereas NMF can never produce a more accurate approximation than the SVD, its proximity to *A* relative to the SVD can be measured. Namely, the relative error, computed as

(5) $$\text{RE}=\frac{{∥A-WH∥}_{F}-{∥A-USV^{T}∥}_{F}}{{∥A-USV^{T}∥}_{F}},$$

where both factorizations are truncated after *k* dimensions (or factors), can show how close the feature vectors produced by the NMF are to the optimal basis [18].

**Figure 1:**
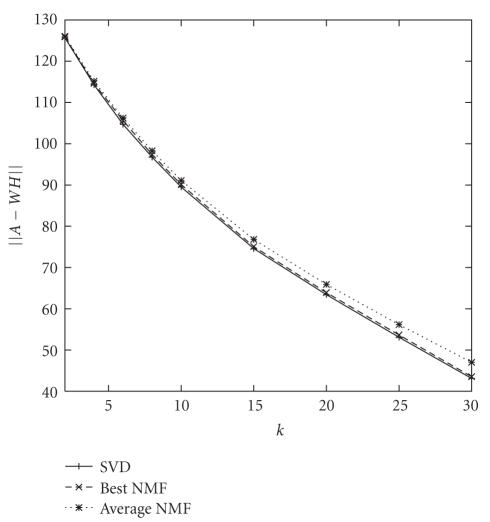
Error measures for the SVD, best NMF run, and average NMF run for the *50TG* collection.

Intuitively, as *k* increases, the NMF factorization should more closely approximate *A*. As shown in Figure 1, this is exactly the case. Surprisingly, however, the average of all converging NMF runs is under 10% relative error compared to the SVD, with that error tending to rise as *k* increases. The proximity of the NMF to the SVD implies that, for this small dataset, NMF can accurately approximate the data.

Next, several different initialization methods (discussed in Section 3.1) were examined. To study the effects on convergence, one set of NMF parameters must be chosen as the baseline against which to compare. By examining the NMF with no additional constraints, the NNDSVDa initialization method consistently produces the most accurate approximation when compared to NNDSVDe, NNDSVDme, NNDSVDz, and random initialization [7]. The relative error NNDSVDa generates less than 1% for most tested values of *k*. Unfortunately, NNDSVDa requires several hundred iterations to converge.

**Figure 2:**
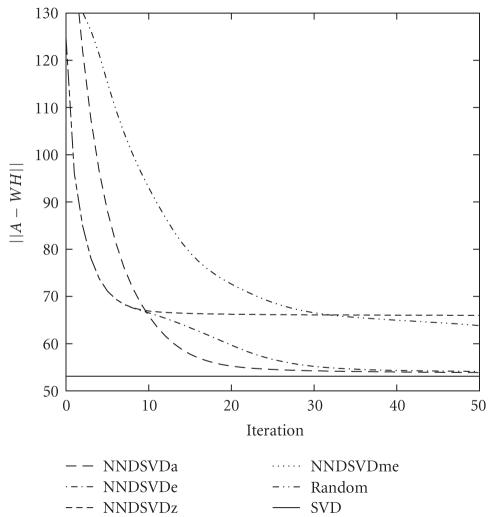
Convergence graph comparing the NNDSVDa, NNDS VDe, NNDSVDme, NNDSVDz, and best random NMF runs of the *50TG* collection for (*k* = 25).

NNDSVDe performs comparably to NNDSVDa with regard to relative error, often within a fraction of a percent. For smaller values of *k*, NNDSVDe takes significantly longer time to converge than NNDSVDa although the exact opposite is true for the larger value of *k*. NNDSVDz, on the other hand, converges much faster for smaller values of *k* at the cost of accuracy as the locked zero elements have an adverse effect on the best solution that can be converged upon. Not surprisingly, NNDSVDme performed comparably to NNDSVDz in many cases, however, it was able to achieve slightly more accurate approximations as the number of iterations increased. In fact, NNDSVDme was identical to NNDSVDz in most cases and will not be mentioned henceforth unless noteworthy behavior is observed. Random initialization performs comparably to NNDSVDa in terms of accuracy and favorably in terms of speed for small *k*, but as *k* increases, both speed and accuracy suffer. A graph illustrating the convergence rates when *k* = 25 is depicted in Figure 2.

**Figure 3:**
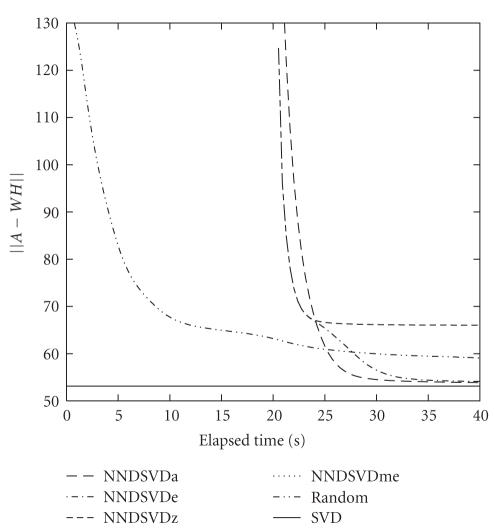
Convergence graph comparing the NNDSVDa, NNDSVDe, NNDSVDme, NNDSVDz, and best random NMF runs of the *50TG* collection for (*k* = 25) taking into account initialization time.

In terms of actual elapsed time, the improved performance of the NNDSVD does not come without a cost. In the context of SGO, the time spent computing the initial SVD of *A* for the first step of the NNDSVD algorithm is assumed to be zero since the SVD is needed a priori for querying purposes However, the initialization time required to complete the NNDSVD when *k* = 25 is nearly 21 seconds, while the cost for random initialization is relatively negligible. All runs were performed on a machine running Debian Linux 3.0 with an Intel Pentium III 1-GHz processor and 256-MB memory. Since the cost per each NMF iteration is nearly.015 seconds per *k* (when *k* = 25), the cost of performing the NNDSVD is (approximately) equivalent to 55 NMF iterations. Convergence taking into account this cost is shown in Figure 3.

### 3.3. Labeling recall

Measuring recall is a quantitative way to validate “known” information within a hierarchy. Here, a method was developed to measure recall at various branch points in a hierarchical tree (described in Section 2.3). The gold standard used for measuring recall included the MeSH headings associated with gene abstracts. The *mean average recall* (MAR) denotes the value attained when the average recall at each level is averaged across all branches of the tree. Here, a hierarchy level refers to all nodes that share the same distance (number of edges) from the root. This section discusses the parameter settings that provided the best labelings, both in the local and global sense to the tree generated in [2] with 47 interior nodes spread across 11 levels.

After applying the labeling algorithm described in Section 2.2 to the factors produced by NMF, the MAR generated was very low (under 25%). Since the NMF-generated vocabulary did not overlap well with the MeSH dictionary, the NMF features were mapped into MeSH features via the procedure outlined in Algorithm 1, where the most dominant feature represented each document only if the corresponding weight in the *H* matrix was greater than 0.5. Also, the top 10 MeSH headings were chosen to represent each document, and the top 100 corresponding terms were extracted to formulate each new MeSH feature vector. Consequently, the resulting MeSH feature vectors produced labelings with greatly increased MAR.

With regard to the accuracy of the labelings, several trends exist. As *k* increases, the achieved MAR increases as well. This behavior could be predicted since increasing the number of features also increases the size of the effective labeling vocabulary, thus enabling a more robust labeling. When *k* = 25, the average MAR across all runs is approximately 68%.

**Figure 4:**
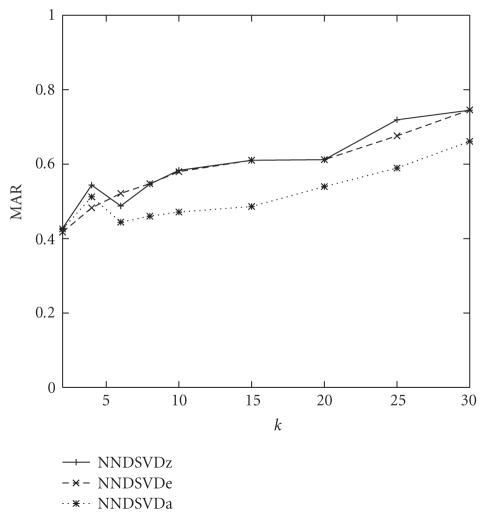
MAR as a function of *k* under the various NNDSVD initialization schemes with no constraints for the *50TG* collection.

Since the NNDSVDa initialization provided the best convergence properties, it will be used as a baseline against which to compare. If *k* is not specified, assume *k* = 25. In terms of MAR, NNDSVDa produced below average results, with both NNDSVDe and NNDSVDz consistently outperforming NNDSVDa for most values of *k*; NNDSVDe and NNDSVDz attained similar MAR values as depicted in Figure 4. The recall of the baseline case using NNDSVDa and *k* = 25 depicted by node level is shown in Figure 6.

**Figure 5:**
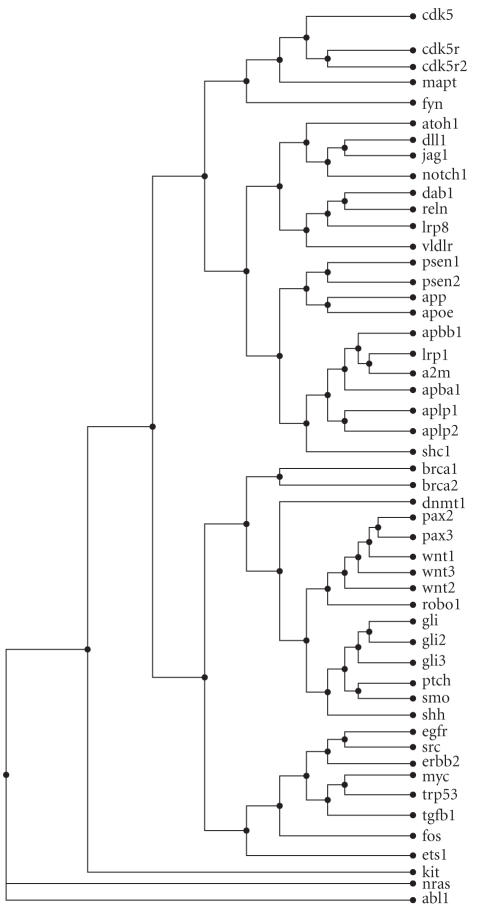
Hierarchical tree for a 50 test gene (50TG) collection described in [2] using updated MEDLINE abstracts.

The 11 node levels of the 50TG hierarchical tree [2] shown in Figure 5 can be broken into thirds to analyze the accuracy of a labeling within a depth region of the tree. The MAR for NNDSVDa for each of the thirds is approximately 58%, 63%, and 54%, respectively. With respect to the topmost third of the tree, any constraint applied to any NNDSVD initialization other than smoothing *W* applied to NNDSVDa provided an improvement over the 58% MAR. In all cases, the resulting MAR was at least 75%. NNDSVDa performed slightly below average over the middle third at 63%. Overall, nearly any constraint improved or matched recall over the base case over all thirds with the exception that enforcing sparsity on *H* underperformed NNDSVDa in the bottom third of the tree; all other constraints achieved at least 54% MAR for the bottom third.

**Figure 6:**
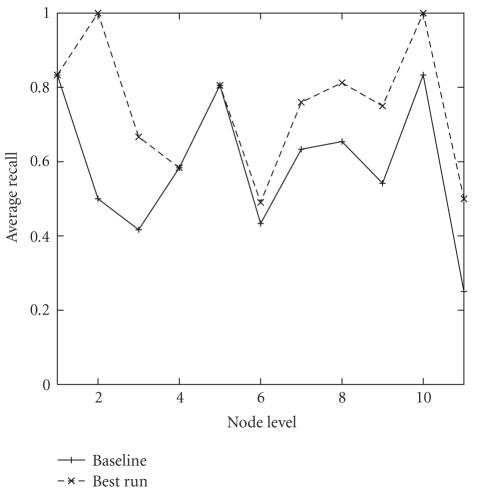
Recall as a function of node level for the NNDSVD initialization on the *50TG* collection. The achieved MAR for the baseline case is 58.95%, while the best achieved MAR for the NNDSVD initialization is 74.56%.

With respect to different values of *k*, similar tendencies exist over all thirds. NNDSVDa is among the worst in terms of MAR with the exception that it does well in the topmost third when *k* is either 2 or 4. There was no discernable advantage when comparing NNDSVD initialization to its random counterpart. Overall, the best NNDSVD (and hence reproducible) MAR was achieved using NNDSVDe and *k* = 30 (also shown in Figure 6).

### 3.4. Labeling evaluation

**Figure 7:**
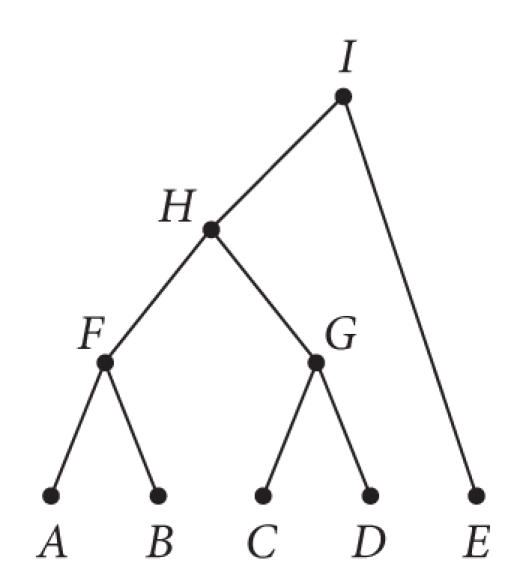
A hierarchical tree containing a set of genes related to Alzheimer's disease (leaf nodes A and B), brain development (leaf nodes C and D), or both Alzheimer's disease and brain development (leaf node E).

Although relative error and recall are measures that can automatically evaluate a labeling, ultimately the final evaluation still requires some manual observation and interpretation. For example, assuming the tree given in Figure 7 with leaf nodes representing the gene clusters given in Table 8, one possible labeling using MeSH headings generated from Algorithm 1 is given in Table 9, and a sample NMF-generated labeling is given in Table 10.

As expected, many of the MeSH terms were too general and were also associated with many of the 5 gene clusters, for example, genetics, proteins, chemistry, and cell. However, some MeSH terms were indeed useful in describing the function of the gene clusters. For example, Cluster A MeSH labels are suggestive of LDL and alpha macroglobulin receptor protein family; Cluster B MeSH labels are associated with Alzheimer's disease and Amyloid beta metabolism; Cluster C labels are associated with extracellular matrix and cell adhesion; Cluster D labels are associated with embryology and inhibotrs; and Cluster E labels are associated with tau protein and lymphocytes.

In contrast to MeSH labeling, the text labeling by NMF was much more specific and functionally descriptive. In general, the first few terms (highest ranking terms) in each cluster defined either the gene name or alias. Interestingly, each cluster also contained terms that were functionally significant. For example, rap (Cluster A) is known to be a ligand for a2m and lrp1 receptors. In addition, the 4 genes in Cluster C are known to be part of a molecular signaling pathway involving Cajal-retzius cells in the brain that control neuronal positioning during development. Lastly, the physiological effects of Notch1 (Cluster D) have been linked to activation of intracellular transcription factors Hes1 and Hes5.

Importantly, the specific nature of text labeling by NMF allows identification of previously unknown functional connections between genes and clusters of genes. For example, the term PS1 appeared in both Cluster B and Cluster D. This finding is very interesting in that PS1 encodes a protein which is part of a protease complex called gamma secretases. In addition to cleaving the Alzheimer protein APP, gamma secretases have been shown to cleave the developmentally important Notch protein. Therefore, these results indicate that NMF labeling provides a useful tool for discovering new functional associations between genes in a cluster as well as across multiple gene clusters.

## 4. DISCUSSION

While comparing NMF runs, several trends can be observed both with respect to mathematical properties and recall tendencies. First, and as expected, as *k* increases, the approximation achieved by the SVD with respect to *A* is more accurate; the NMF can provide a relatively close approximation to *A* in most cases, but the error also increases with *k*. Second, NNDSVDa provides the fastest convergence in terms of number of iterations to the closest approximations. Third, applying additional constraints such as smoothing and sparsity [7] has little noticeable effect on both convergence and recall, and in many cases greatly decreases the likelihood that a stationary point will be reached. Finally, to generate relatively “good” approximation error (within 5%), about 20–40 iterations are recommended using either NNDSVDa or NNDSVDe initialization with no additional constraints when *A* is reasonably large (about half the number of documents). For smaller *k*, performing approximately 25 iterations under random initialization will usually accomplish 5% relative error, with the number of iterations required decreasing as *k* decreases.

While measuring error norms and convergence is useful to expose mathematical properties and structural tendencies of the NMF, the ultimate goal of this application is to provide a useful labeling of a hierarchical tree from the NMF. In many cases, the “best” labeling may be provided by a suboptimal run of NMF. Overall, more accurate labelings resulted from higher values of *k* because more feature vectors increased the vocabulary size of the labeling dictionary. Generally speaking, the NNDSVDe, NNDSVDme, and NNDSVDz schemes outperformed the NNDSVDa initialization. Overall, the accuracy of the labelings appeared to be more a function of *k* and the initial seed rather than the constraints applied.

Much research is being performed concerning the NMF, and this work examines three methods based on the multiplicate update (see Section 2.1). Many other NMF variations exist and more are being developed, so their application to the biological realm should be studied. For example, [19] proposes a hybrid least squares approach called GD-CLS to solve NMF and overcomes the problem of “locking” zeroed elements encountered by MM, [20, 21] propose nonsmooth NMF as an alternative method to incorporate sparseness, and [22] proposes an NMF technique that generates three factor matrices and has shown promising clustering results. NMF has been applied to microarray data [23], but efforts need to be made to combine the text information with microarray data; some variation of tensor factorization could possibly show how relationships change over time [24].

With respect to labeling methods, MeSH heading labels were generally useful, but provided little specific details about the functional relationship between the genes in a cluster. On the other hand, text labeling provided specific and detailed information regarding the function of the genes in a clusters. Importantly, term labels provided some specific connections between groups of genes that were not readily apparent. Thus, term labeling offers a distinct advantage for discovering new relationships between genes and can aid in interpretation of high throughput data.

Regardless of the techniques employed, one of the issues that will always be prevalent regarding biological data is that of quality versus quantity. Inherently related to this problem is the establishment of standards within the field especially as they pertain to hierarchical data. Efforts such as gene ontology (GO) are being built and refined [25], but standard datasets for comparing results and clearly defined (and accepted) evaluation measures could facilitate more meaningful comparisons between methods.

In the case of SGO, developing methods to derive “known” data is a major issue (even GO does not produce a “gold standard” hierarchy given a set of genes). Access to more data and to other hierarchies would help test the robustness of the method, but that remains one of the problems inherent in the field. In general, approximations that are more mathematically optimal do not always produce the “best” labeling. Often, factorizations provided by the NMF can be deemed “good enough,” and the final evaluation will remain subjective. In the end, if automated approaches can approximate that subjectivity, then greater understanding of more data will result.

## Figures and Tables

**Algorithm 1:**
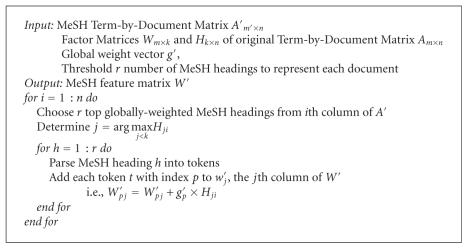
Feature vector replacement algorithm.

**Table 1:** Term-document matrix for the sample collection in Table 2.

	d1	d2	d3	d4	d5	d6	d7	d8	d9
Alcoholism	—	0.4338	—	—	—	0.2737	—	0.2737	0.4338
Anxiety	0.4745	—	—	—	0.4745	—	—	—	—
Attack	—	—	—	—	0.6931	—	—	—	—
Autism	—	—	—	—	—	—	0.7520	—	0.7520
Airth	—	—	—	—	—	0.4745	—	—	0.4745
Blood	—	—	—	0.3466	0.3466	0.3466	—	—	—
Bone	—	—	0.7520	0.7520	—	—	—	—	—
Cancer	—	0.4745	0.4745	—	—	—	—	—	—
Cells	—	—	—	0.6931	—	—	—	—	—
Children	—	—	—	—	—	—	0.4745	—	0.4745
Cirrhosis	—	0.7520	—	—	—	—	—	0.7520	—
Damage	—	—	0.6931	—	—	—	—	—	—
Defects	—	—	—	—	—	0.3466	0.3466	—	0.3466
Failure	—	0.4745	—	—	—	0.4745	—	—	—
Hypertension	—	—	—	—	—	0.6931	—	—	—
Kidney	—	0.4745	—	—	—	0.4745	—	—	—
Leukemia	—	—	1.0986	—	—	—	—	—	—
Liver	—	0.4745	—	—	—	—	—	0.4745	—
Marrow	—	—	0.7520	0.7520	—	—	—	—	—
Pressure	—	—	—	—	0.7804	0.4923	—	—	—
Scarring	—	—	—	—	—	—	—	0.6931	—
Speech	—	—	—	—	—	—	0.6931	—	—
Stress	0.4923	—	—	—	0.7804	—	—	—	—
Tuberculosis	—	—	—	0.6931	—	—	—	—	—

**Table 2:** Sample collection with dictionary terms displayed in *bold*.

Document	Text
d1	Work-related *stress* can be considered a factor contributing to *anxiety*.
d2	*Liver cancer* is most commonly associated with *alcoholism* and *cirrhosis*. It is well-known that *alcoholism* can cause *cirrhosis* and increase the risk of *kidney failure*.
d3	*Bone marrow* transplants are often needed for patients with *leukemia* and other types of *cancer* that *damage bone marrow*. Exposure to toxic chemicals is a risk factor for *leukemia*.
d4	Different types of *blood cells* exist in *bone marrow*. *Bone marrow* procedures can detect *tuberculosis*.
d5	Abnormal *stress* or *pressure* can cause an *anxiety attack*. Continued *stress* can elevate *blood pressure*.
d6	*Alcoholism* can cause high *blood pressure* (*hypertension*) and increase the risk of *birth defects* and *kidney failure*.
d7	The presence of *speech defects* in *children* is a sign of *autism*. As of yet, there is no consensus on what causes *autism*.
d8	*Alcoholism*, often triggered at an early age by factors such as environment and genetic predisposition, can lead to *cirrhosis*. *Cirrhosis* is the *scarring* of the *liver*.
d9	*Autism* affects approximately 0.5% of *children* in the US. The link between *alcoholism* and *birth defects* is well-known; researchers are currently studying the link between *alcoholism* and *autism*.

**Table 3:** Feature matrix *W* for the sample collection.

	f1	f2	f3	f4
Alcoholism	0.0006	0.3503	—	—
Anxiety	—	—	0.4454	—
Attack	—	—	0.4913	—
Autism	—	0.0030	—	0.8563
Birth	—	0.1111	0.0651	0.2730
Blood	0.0917	0.0538	0.3143	—
Bone	0.5220	—	0.0064	—
Cancer	0.1974	0.1906	—	—
Cells	0.1962	—	0.0188	—
Children	—	0.0019	—	0.5409
Cirrhosis	0.0015	0.5328	—	—
Damage	0.2846	—	—	—
Defects	—	0.0662	—	0.4161
Failure	0.0013	0.2988	—	—
Hypertension	—	0.1454	0.1106	—
Kidney	0.0013	0.2988	—	—
Leukemia	0.4513	—	—	—
Liver	0.0009	0.3366	—	—
Marrow	0.5220	—	0.0064	—
Pressure	—	0.066	0.6376	—
Scarring	—	0.208	—	—
Speech	—	—	—	0.4238
Stress	—	—	0.6655	—
Tuberculosis	0.1962	—	0.0188	—

**Table 4:** Coefficient matrix *H* for the sample collection.

	d1	d2	d3	d4	d5	d6	d7	d8	d9
f1	—	0.0409	1.6477	1.1382	0.0001	0.0007	—	—	—
f2	—	1.3183	—	—	0.0049	0.6955	0.0003	0.9728	0.2219
f3	0.3836	—	—	0.0681	1.1933	0.3327	—	—	—
f4	—	—	—	—	—	0.1532	0.9214	—	0.799

**Table 5:** Approximation to sample term-document matrix given in Table 1.

	d1	d2	d3	d4	d5	d6	d7	d8	d9
Alcoholism	—	0.4618	0.0010	0.0007	0.0017	0.2436	0.0001	0.3408	0.0777
Anxiety	0.1708	—	—	0.0303	0.5315	0.1482	—	—	—
Attack	0.1884	—	—	0.0334	0.5863	0.1635	—	—	—
Autism	—	0.0040	—	—	—	0.1333	0.7890	0.0029	0.6848
Birth	0.0250	0.1464	—	0.0044	0.0783	0.1407	0.2516	0.1080	0.2428
Blood	0.1206	0.0746	0.1511	0.1258	0.3754	0.1420	—	0.0523	0.0119
Bone	0.0025	0.0214	0.8602	0.5946	0.0077	0.0025	—	—	—
Cancer	—	0.2593	0.3252	0.2247	0.001	0.1327	0.0001	0.1854	0.0423
Cells	0.0072	0.0080	0.3233	0.2246	0.0224	0.0064	—	—	—
Children	—	0.0025	—	—	—	0.0842	0.4984	0.0019	0.4326
Cirrhosis	—	0.7025	0.0024	0.0017	0.0026	0.3705	0.0002	0.5183	0.1183
Damage	—	0.0116	0.4689	0.3239	—	0.0002	—	—	—
Defects	—	0.0873	—	—	0.0003	0.1098	0.3834	0.0644	0.3472
Failure	—	0.3939	0.0022	0.0015	0.0015	0.2078	0.0001	0.2906	0.0663
Hypertension	0.0424	0.1916	—	0.0075	0.1327	0.1379	—	0.1414	0.0323
Kidney	—	0.3939	0.0022	0.0015	0.0015	0.2078	0.0001	0.2906	0.0663
Leukemia	—	0.0185	0.7437	0.5137	—	0.0003	—	—	—
Liver	—	0.4437	0.0015	0.0011	0.0017	0.2341	0.0001	0.3274	0.0747
Marrow	0.0025	0.0214	0.8602	0.5946	0.0077	0.0025	—	—	—
Pressure	0.2445	0.0870	—	0.0434	0.7612	0.2580	—	0.0642	0.0147
Scarring	—	0.2742	—	—	0.0010	0.1446	0.0001	0.2023	0.0462
Speech	—	—	—	—	—	0.0649	0.3905	—	0.3386
Stress	0.2553	—	—	0.0453	0.7942	0.2214	—	—	—
Tuberculosis	0.0072	0.0080	0.3233	0.2246	0.0224	0.0064	—	—	—

**Table 6:** Top 5 words for each feature from the sample collection.

f1	f2	f3	f4
Bone	Cirrhosis	Stress	Autism
Marrow	Alcoholism	Pressure	Children
Leukemia	Liver	Attack	Speech
Damage	Kidney	Anxiety	Defects
Cancer	Failure	Blood	Birth

**Table 7:** Rearranged term-document matrix for the sample collection.

	d3	d4	d2	d6	d8	d1	d5	d7	d9
Bone	0.7520	0.7520	—	—	—	—	—	—	—
Cancer	0.4745	—	0.4745	—	—	—	—	—	—
Cells	—	0.6931	—	—	—	—	—	—	—
Damage	0.6931	—	—	—	—	—	—	—	—
Leukemia	1.0986	—	—	—	—	—	—	—	—
Marrow	0.7520	0.7520	—	—	—	—	—	—	—
Tuberculosis	—	0.6931	—	—	—	—	—	—	—

Alcoholism	—	—	0.4338	0.2737	0.2737	—	—	—	0.4338
Cirrhosis	—	—	0.7520	—	0.7520	—	—	—	—
Failure	—	—	0.4745	0.4745	—	—	—	—	0.4745
Hypertension	—	—	—	0.6931	—	—	—	—	—
Kidney	—	—	0.4745	0.4745	—	—	—	—	0.4745
Liver	—	—	0.4745	—	0.4745	—	—	—	—
Scarring	—	—	—	—	0.6931	—	—	—	—

Anxiety	—	—	—	—	—	0.4745	0.4745	—	—
Attack	—	—	—	—	—	—	0.6931	—	—
Blood	—	0.3466	—	0.3466	—	—	0.3466	—	—
Pressure	—	—	—	0.4923	—	—	0.7804	—	—
Stress	—	—	—	—	—	0.4923	0.7804	—	—

Autism	—	—	—	—	—	—	—	0.7520	0.7520
Birth	—	—	—	0.4745	—	—	—	—	0.4745
Children	—	—	—	—	—	—	—	0.4745	0.4745
Defects	—	—	—	0.3466	—	—	—	0.3466	0.3466
Speech	—	—	—	—	—	—	—	0.6931	—

**Table 8:** Genes comprising each leaf node of the tree shown in Figure 7.

A	B	C	D	E
a2m	apoe	dab1	atoh1	cdk5
apba1	app	lrp8	dll1	cdk5r
apbb1	psen1	reln	jag1	cdk5r2
aplp1	psen2	vldlr	notch1	fyn
aplp2	—	—	—	mapt
lrp1	—	—	—	—
shc1	—	—	—	—

**Table 9:** Top 10 MeSH terms for the leaf nodes of the tree shown in Figure 7.

A	B	C	D	E
Metabolism	Protein	Genetics	Genetics	Metabolism
Genetics	Amyloid	Molecules	Proteins	Proteins
Protein	Beta	Neuronal	Metabolism	Genetics
Proteins	Genetics	Adhesion	Membrane	Tau
Receptor	Metabolism	Cell	Cell	Protein
Related	Precursor	Metabolism	Physiology	Lymphocyte
ldl	Chemistry	Proteins	Cytology	p56
Macroglobulins	Apolipoproteins	Extracellular	Embryology	Specific
Alpha	Disease	Matrix	Biosynthesis	lck
Chemistry	Alzheimer	Biosynthesis	Inhibitors	Tyrosine

**Table 10:** Top 10 terms for the leaf nodes of the tree shown in Figure 7.

A	B	C	D	E
lrp	Apoe	reelin	Notch	fyn
Receptor-related	ps1	reeler	notch1	Tau
Lipoprotein	Amyloid	dab1	jagged1	cdk5
fe65	Abeta	vldlr	notch-1	lck
app	Presenilin	apoer2	hes5	sh3
Alpha	Epsilon	Positioning	Fringe	nmda
rap	Apolipoprotein	Cajal-retzius	hes-1	Ethanol
Abeta	Alzheimer	apoe	hes1	Phosphorylation
Beta-amyloid	ad	Apolipoprotein	hash1	Alcohol
Receptor	Gamma-secretase	Lipoprotein	ps1	tcr
